# Diagnostic doses and times for *Phlebotomus papatasi* and *Lutzomyia longipalpis* sand flies (Diptera: Psychodidae: Phlebotominae) using the CDC bottle bioassay to assess insecticide resistance

**DOI:** 10.1186/s13071-016-1496-3

**Published:** 2016-04-15

**Authors:** David S. Denlinger, Joseph A. Creswell, J. Laine Anderson, Conor K. Reese, Scott A. Bernhardt

**Affiliations:** Department of Biology, Utah State University, Logan, Utah USA

**Keywords:** *Lutzomyia longipalpis*, *Phlebotomus papatasi*, Insecticide resistance, CDC, WHO, Bottle bioassay, Pyrethroid, Organophosphate, Carbamate, DDT

## Abstract

**Background:**

Insecticide resistance to synthetic chemical insecticides is a worldwide concern in phlebotomine sand flies (Diptera: Psychodidae), the vectors of *Leishmania* spp. parasites. The CDC bottle bioassay assesses resistance by testing populations against verified diagnostic doses and diagnostic times for an insecticide, but the assay has been used limitedly with sand flies. The objective of this study was to determine diagnostic doses and diagnostic times for laboratory *Lutzomyia longipalpis* (Lutz & Nieva) and *Phlebotomus papatasi* (Scopoli) to ten insecticides, including pyrethroids, organophosphates, carbamates, and DDT, that are used worldwide to control vectors.

**Methods:**

Bioassays were conducted in 1,000-ml glass bottles each containing 10–25 sand flies from laboratory colonies of *L. longipalpis* or *P. papatasi*. Four pyrethroids, three organophosphates, two carbamates and one organochlorine, were evaluated. A series of concentrations were tested for each insecticide, and four replicates were conducted for each concentration. Diagnostic doses were determined only during the exposure bioassay for the organophosphates and carbamates. For the pyrethroids and DDT, diagnostic doses were determined for both the exposure bioassay and after a 24-hour recovery period.

**Results:**

Both species are highly susceptible to the carbamates as their diagnostic doses are under 7.0 μg/ml. Both species are also highly susceptible to DDT during the exposure assay as their diagnostic doses are 7.5 μg/ml, yet their diagnostic doses for the 24-h recovery period are 650.0 μg/ml for *Lu. longipalpis* and 470.0 μg/ml for *P. papatasi*.

**Conclusions:**

Diagnostic doses and diagnostic times can now be incorporated into vector management programs that use the CDC bottle bioassay to assess insecticide resistance in field populations of *Lu. longipalpis* and *P. papatasi*. These findings provide initial starting points for determining diagnostic doses and diagnostic times for other sand fly vector species and wild populations using the CDC bottle bioassay.

## Background

Insecticide resistance continues to be a threat to the success of insect vector control programs that incorporate synthetic chemical insecticides [1]. Insecticide resistance is a heritable phenotype that allows arthropods to survive an exposure to an insecticide that would normally kill a susceptible population [2–4]. Today, insecticide resistance to all classes of synthetic insecticides has been found in the major insect vectors [1, 5]. Managing insecticide resistance requires timely, accurate data through resistance monitoring and insecticide evaluation to assess a vector species’ susceptibility to insecticides. These aspects can be used to develop effective strategies at managing vector populations [6]. The primary way to assess insecticide resistance is to use insecticide susceptibility bioassays.

The Centers for Disease Control and Prevention (CDC) bottle bioassay is one technique used to measure a vector species’ susceptibility to insecticides [7, 8]. This bioassay is an economical and portable alternative to the World Health Organization’s (WHO) exposure kit bioassay, especially in geographic regions where the WHO bioassay cannot be implemented [9–11]. Another benefit of the CDC bottle bioassay is that the materials, including the glass bottles, can be locally acquired and prepared on site [12].

Sand flies (Diptera: Psychodidae: Phlebotominae) require resistance monitoring because they have been, and continue to be, actively targeted with insecticides [13–16]. Fewer than seventy species of sand flies, including *Lutzomyia longipalpis* (Lutz & Nieva) and *Phlebotomus papatasi* Scopoli, are capable of vectoring *Leishmania* spp. parasites, infection with which causes leishmaniasis, a world-wide disease currently infecting millions of people [17, 18]. Sand fly populations around the world have been exposed to the four main classes of insecticides: organochlorines, organophosphates, carbamates and pyrethroids. Insecticide exposure has been both intentional in directed vector control efforts and inadvertent as part of vector control efforts targeted against other insects [6, 13, 17, 19–24]. Populations of sand flies have been found to be tolerant or resistant, using the WHO exposure kit bioassay and diagnostic doses derived for mosquitoes, to the insecticides used worldwide [6, 19–30]. Despite these examples, there is a gap in understanding the prevalence of insecticide resistance in sand fly populations. This has been attributed to challenges in collecting the necessary number of live flies for the bioassays and because there is a lack of a standardized sand fly bioassay [31].

To test an insect vector species’ susceptibility status to an insecticide using the CDC bottle bioassay, a diagnostic dose and diagnostic time are needed for that insecticide [8]. A diagnostic dose is the lowest dose of an insecticide that causes 100 % mortality in a susceptible population between 30 and 60 minutes, the diagnostic time [8]. There have been few published studies that have determined diagnostic doses for phlebotomine sand flies using the CDC bottle bioassay. In Colombia, Santamaría et al. [32] determined the diagnostic dose of lambda(λ)-cyhalothrin to be 10.0 μg/ml for *Lu. longipalpis*. One concern with this finding is that Santamaría et al. [32] only tested three concentrations of lambda(λ)-cyhalothrin (10.0, 50.0, and 100.0 μg/ml), which makes it difficult to identify a precise diagnostic dose and diagnostic time because of the large differences between the doses tested [33]. Also working with *Lu. longipalpis*, Marceló et al. [33] determined the diagnostic doses and diagnostic times for malathion, deltamethrin, and lambda(λ)-cyhalothrin to be 75.0 μg/ml in 25 minutes, 10.0 μg/ml in 35 minutes and 15.0 μg/ml in 30 minutes, respectively. Diagnostic doses and diagnostic times for field-collected *Lu. evansi*, an important vector of *Le. infantum* in the Americas, have been previously described as 7.0 μg/ml in 10 minutes for deltamethrin and 3.5 μg/ml in 10 minutes for lambda(λ)-cyhalothrin [20].

Dose–response survival curves to determine lethal concentrations causing 50 %, 90 % and 95 % mortality for laboratory colonies of *Lu. longipalpis* and *P. papatasi* to ten insecticides were previously determined using a modified version of the CDC bottle bioassay and the WHO exposure kit [34]. These concentrations can serve as starting points for determining diagnostic doses and diagnostic times from time-response survival curves for a susceptible population of any sand fly species. Recently, Li et al. [31] also describes a bottle bioassay using 20 ml glass scintillation vials to determine lethal times causing 50 % mortality for *P. papatasi* and *P. duboscqi* exposed to ten pyrethroid and organophosphate insecticides. While not diagnostic doses, these data can be used for comparative purposes for future insecticide resistance studies for *P. papatasi* and *P. duboscqi*, two important Old World *Leishmania* spp. vectors.

The objective of this study is to define and establish diagnostic doses and diagnostic times using the CDC bottle bioassay for *Lu. longipalpis* and *P. papatasi* to ten insecticides. No standardized diagnostic doses exist for insecticides using the CDC bottle bioassay. These diagnostic doses and diagnostic times determined in this study can now be incorporated into future studies assessing insecticide resistance from field-collected sand fly populations.

## Methods

### Sand flies

Laboratory strains of insecticide-susceptible *Lu. longipalpis* and *P. papatasi* sand flies at Utah State University were derived from 30-year established colonies maintained at the Walter Reed Army Institute of Research (WRAIR) (Silver Spring, MD, USA). The original colonies from Walter Reed have never been exposed to insecticides. All life stages were reared and maintained at USU [34–38].

### Insecticides

Ten technical-grade insecticides were used in this study: four pyrethroids [cypermethrin (Sigma-Aldrich, St. Louis, MO, USA), deltamethrin (Sigma-Aldrich, St. Louis, MO, USA), lambda(λ)-cyhalothrin (Sigma-Aldrich, St. Louis, MO, USA), and permethrin (Chem Service, Inc., West Chester, PA, USA)]; three organophosphates [chlorpyrifos (Sigma-Aldrich, St. Louis, MO, USA), fenitrothion (Sigma-Aldrich, St. Louis, MO, USA), and malathion (Chem Service, Inc., West Chester, PA, USA)]; two carbamates [bendiocarb (Sigma-Aldrich, St. Louis, MO, USA) and propoxur (Sigma-Aldrich, St. Louis, MO, USA)]; and the organochlorine dichlorodiphenyltrichloroethane (DDT) (Sigma-Aldrich, St. Louis, MO, USA). All insecticide dilutions were prepared in acetone, stored in glass bottles, wrapped in aluminum foil, and kept at 4 °C while not being used [8]. The concentrations of each insecticide used in these experiments are listed in (Table 1). Whole-value lethal concentrations causing 50 % and 90 % mortality for each insecticide and for each sand fly species from Denlinger et al. [34] were used as initial concentrations tested for determining diagnostic doses.

**Table 1:** Concentrations of ten insecticides used to expose *Lu. longipalpis* and *P. papatasi* sand flies

Insecticide (Insecticide class^a^)	Species	Concentration (μg insecticide/bottle)
Cypermethrin (PYR)	*Lu. longipalpis*	5.0, 10.0, 15.0, 20.0
	*P. papatasi*	20.0, 25.0, 30.0, 35.0, 40.0, 45.0, 50.0, 55.0, 60.0, 65.0, 70.0, 75.0, 90.0, 95.0
Deltamethrin (PYR)	*Lu. longipalpis*	5.0, 10.0, 15.0, 20.0, 25.0, 30.0, 35.0, 40.0, 45.0, 50.0, 75.0, 100.0
	*P. papatasi*	5.0, 10.0, 15.0, 20.0, 25.0, 30.0, 35.0, 40.0, 50.0, 75.0, 100.0
λ-Cyhalothrin (PYR)	*Lu. longipalpis*	1.0, 2.0, 3.0, 4.0, 10.0, 20.0, 30.0, 40.0
	*P. papatasi*	1.0, 2.0, 3.0, 4.0, 5.0, 6.0, 7.0, 10.0, 20.0, 30.0, 40.0
Permethrin (PYR)	*Lu. longipalpis*	5.0, 10.0, 12.5, 15.0, 20.0
	*P. papatasi*	10.0, 20.0, 25.0, 30.0, 35.0, 40.0, 45.0, 50.0
Chlorpyrifos (OP)	*Lu. longipalpis*	5.0, 10.0, 15.0, 20.0, 25.0, 30.0
	*P. papatasi*	20.0, 25.0, 30.0, 35.0, 40.0, 45.0
Fenitrothion (OP)	*Lu. longipalpis*	2.0, 4.0, 6.0, 8.0, 10.0, 12.0, 14.0, 16.0, 18.0, 20.0, 22.0, 24.0, 26.0, 28.0, 30.0, 32.0
	*P. papatasi*	5.0, 10.0, 15.0, 20.0, 25.0, 30.0, 35.0, 40.0, 45.0
Malathion (OP)	*Lu. longipalpis*	5.0, 10.0, 15.0, 20.0, 25.0, 30.0, 35.0, 40.0, 45.0
	*P. papatasi*	50.0, 75.0, 100.0, 125.0, 130.0, 135.0, 140.0, 145.0
Bendiocarb (CX)	*Lu. longipalpis*	1.0, 2.0, 3.0, 4.0, 5.0, 6.0, 7.0, 8.0
	*P. papatasi*	1.0, 2.0, 3.0, 4.0, 5.0, 6.0,
Propoxur (CX)	*Lu. longipalpis*	1.0, 2.0, 3.0, 4.0, 10.0
	*P. papatasi*	1.0, 2.0, 3.0, 7.0, 15.0
DDT (OC)	*Lu. longipalpis*	2.5, 5.0, 7.5, 10.0, 15.0, 20.0, 50.0, 100.0, 150.0, 200.0, 250.0, 300.0, 350.0, 400.0, 450.0, 500.0, 550.0, 600.0, 630.0, 635.0, 640.0, 645.0, 650.0, 700.0
	*P. papatasi*	2.5, 5.0, 7.5, 10.0, 50.0, 100.0, 150.0, 200.0, 350.0, 400.0, 450.0, 455.0, 460.0, 465.0, 470.0, 480.0, 490.0, 500.0, 550.0

^a^PYR, pyrethroid; OP, organophosphate; CX, carbamate; OC, organochlorine

### Preparation of exposure bottles

The day before exposing the sand flies, four 1,000-ml glass bottles (Fisher Scientific, Pittsburgh, PA, USA) were prepared by coating them with insecticide, as described in Denlinger et al. [34]. Following Brogdon & Chan [8] for a 250-ml bottle, 1.0 ml of insecticide at 10.0 μg insecticide/ ml acetone gives a concentration of 10.0 μg/ 250-ml bottle. To compensate for these larger bottle sizes and to maintain an equivalence of X μg insecticide/ 250-ml bottle [8], 4.0 ml of X μg insecticide was used to coat the interior of the 1,000-ml bottle [34]. The bottles were coated with insecticide by swirling the acetone: insecticide solution on the bottom, on the sides, and on the lid. The bottle was then placed on a mechanical bottle roller for 30 minutes to dry and reduce the potential for bubble formation. During this time, the lids were slowly loosened to allow the acetone to evaporate. After 30 minutes, the caps were removed, and the bottles were rolled until all of the acetone evaporated. The bottles were then left open to dry overnight in the dark to prevent photodegradation of the insecticides. For each test replicate, one bottle serving as a control was coated with 4.0 ml of acetone [8]. All bottles were re-used throughout the duration of the experiment. To clean a bottle with residual insecticide, the bottle and lid was first triple-rinsed with acetone; filled with warm, soapy water; drained; rinsed and filled with cold water; drained; and autoclaved for at least 20 minutes. After being autoclaved, the bottles were left to dry for at least one day before being used again [34].

### Insecticide exposure tests

Approximately 12 hours after the bottles were prepared with insecticide, 10–25 adult sand flies at least two days post-eclosion were aspirated from the main colony and gently blown into each bottle [8]. Approximately equal numbers of nulliparous female and male flies were used for each insecticide-coated bottle, while only nulliparous females were used in the control bottle [8]. Sand flies were aspirated into the control bottle first, followed by the four insecticide-coated bottles. Once sand flies had been aspirated into all five bottles, the timer was initiated and recorded as time zero. At time zero, the total number of flies in each bottle was recorded. The number of alive or dead sand flies was recorded at each time point, depending on which was easier to visually determine [8]. All bottles were held horizontally for the duration of the experiment. During initial replicates with the largest doses of DDT, the authors infrequently observed that the legs of some sand flies would become stuck to the interior surface of the bottles during the 60-minute exposure. These flies were unable to be removed from the bottles via aspiration. These replicates were not used. To remedy this issue at these high concentrations, the bottles were rotated every few minutes to promote limited hopping and movement of the sand flies. This movement reduced extended surface contact in one place and eliminated the issue of sand flies becoming fixed on the insecticide surface.

The percent mortality at each time point was the average of the percent mortalities of the four replicates. The percent mortality at a time point in the insecticide-treated bottles was corrected with Abbott’s formula if mortality in the control bottle ranged between 5 and 20 %. Abbott’s formula was not used to correct experimental mortalities if the control group mortality was less than 5 %. If control group mortalities exceeded 20 %, the entire testing replicate was not used [24].

#### Organophosphates and carbamates

Mortality was recorded at 0, 15, 30, 35, 40, 45, 60, 75, 90, 105 and 120 minutes by gently rotating the bottle (time-to-knockdown) [8]. Sand flies were scored as “dead” if they had difficulty flying, could not fly altogether, or had trouble righting themselves [8]. If all sand flies were scored as dead before 120 minutes, the flies were kept in the bottles and continued to be observed until 120 minutes was reached.

#### Pyrethroids and DDT

Mortality was scored during the exposure test (time-to-knockdown) to create survival curves as well after 24-hours of recovery time (24-h mortality) [8]. During the exposure test, mortality was recorded at 0, 15, 30, 35, 40, 45 and 60 minutes by gently rotating the bottle. Scoring mortality was equivalent to the criteria used for the carbamate and organophosphate insecticides. If all sand flies were scored as dead before 60 minutes, the flies were kept in the bottles until 60 minutes was reached. At the end of the 60 minutes, the sand flies were captured via mechanical aspiration, released into 1-pint cardboard containers with a fine mesh screen top, and kept under the same environmental and food source conditions as the main, untreated colonies. Sand flies were held in these containers for 24-hours prior to mortality being recorded. Mortality was corrected with Abbott’s formula using the same criteria described above for both the time-to-knockdown and 24-h mortality.

### Survival curves

Time-response survival curves were made for each insecticide for each sand fly species by plotting time on the X-axis against percent mortality on the Y-axis [8]. For each insecticide dose, the percent mortality at each time point is the average mortality between all four insecticide-treated bottles. A diagnostic dose was determined to be the lowest dose tested that caused 100 % mortality between 30 and 60 minutes, the diagnostic time [8].

## Results

**Fig. 1:**
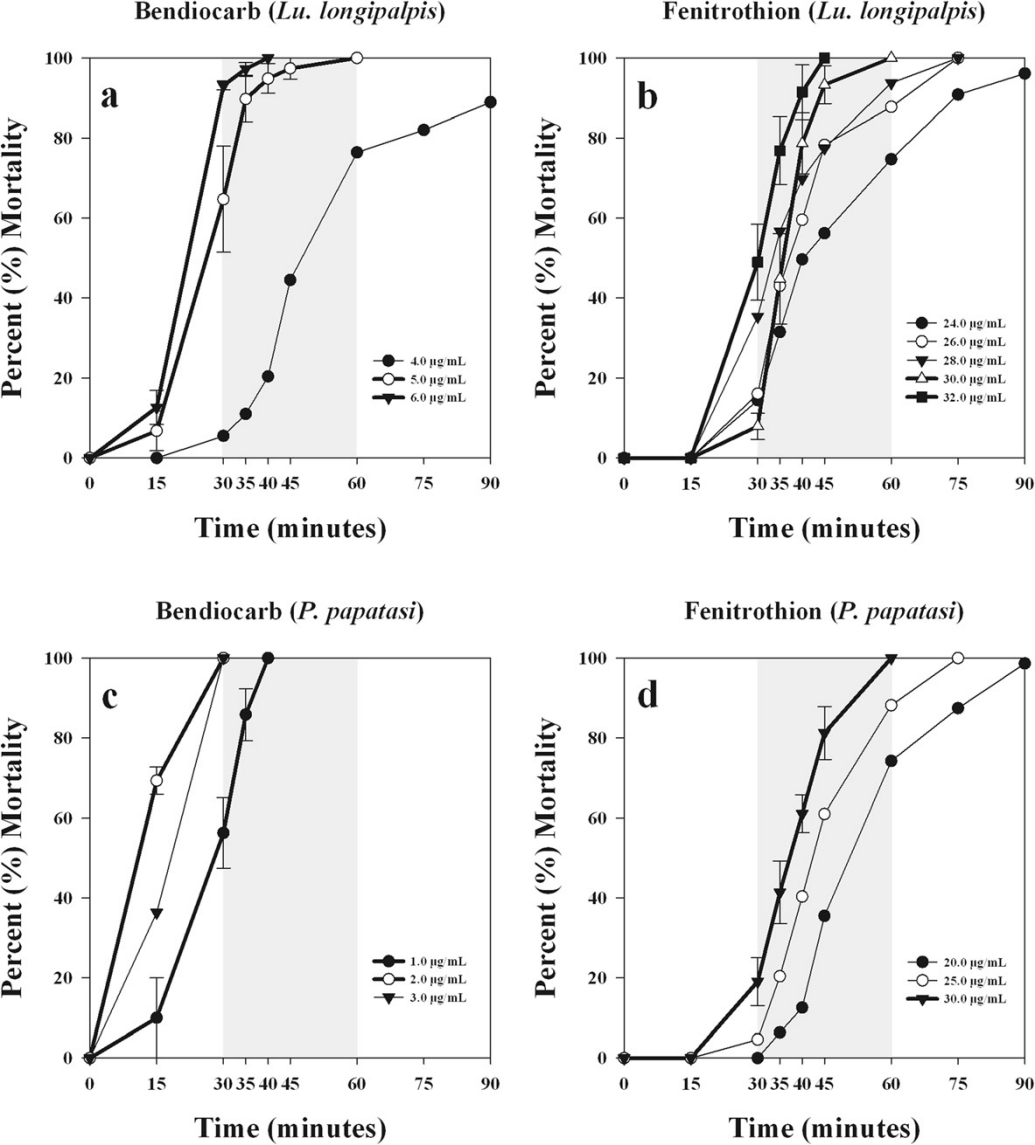
Time-to-knockdown survival curves for *Lu. longipalpis* to bendiocarb (**a**) and fenitrothion (**b**) and for *P. papatasi* to bendiocarb (**c**) and fenitrothion (**d**). For each graph, the thick lines represent the time-response for doses that are considered diagnostic doses. At each time point of the thick lines the error bars show the standard error of the mean percent mortality, across the four bottle replicates. Error bars are only displayed on the diagnostic dose lines for visual clarity. The shaded region of each graph designates a window of time (30, 35, 40, 45 or 60 minutes) that can be considered diagnostic times for diagnostic doses

**Fig. 2:**
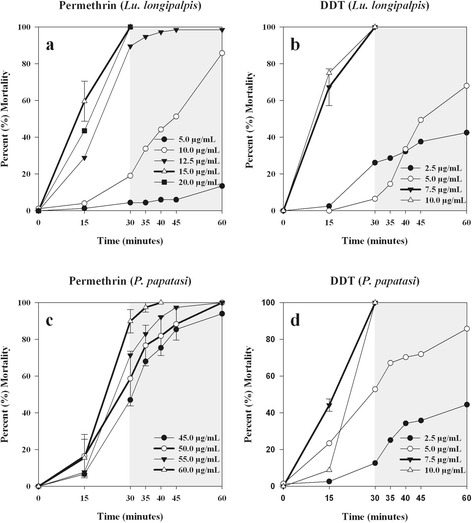
Time-to-knockdown survival curves for *Lu. longipalpis* to permethrin (**a**) and DDT (**b**) and for *P. papatasi* to permethrin (**c**) and DDT (**d**). For each graph, the thick lines represent the time-response for doses that are considered diagnostic doses. At each time point of the thick lines the error bars show the standard error of the mean percent mortality, across the four bottle replicates. Error bars are only displayed on the diagnostic dose lines for visual clarity. The shaded region of each graph designates a window of time (30, 35, 40, 45 or 60 minutes) that can be considered diagnostic times for diagnostic doses

A time-response survival curve for each of the ten insecticides for both *Lu. longipalpis* and *P. papatasi* was created following Brogdon & Chan [8]. For all the time-to-knockdown survival curves, the time to reach 100 % mortality decreased with increasing insecticide concentrations. Diagnostic doses and diagnostic times for the organophosphates and carbamates are presented in (Table 2). Diagnostic doses and diagnostic times for time-to-knockdown and for 24-h mortality for the pyrethroids and DDT are presented in (Table 3). Representative survival curves for bendiocarb, fenitrothion, permethrin, and DDT are presented in (Figs. 1 and 2). For some insecticides, multiple diagnostic doses and diagnostic times were observed. Whereas for other insecticides, only one diagnostic dose and diagnostic time were observed because all of the other doses that were tested for that specific insecticide either did not cause 100 % mortality between 30 and 60 minutes or they were saturated doses.

**Table 2:** Diagnostic doses and diagnostic times for organophosphate and carbamate insecticides at the time-to-knockdown

Insecticide (Insecticide class^a^)	Species	Diagnostic dose and diagnostic time (for time-to-knockdown)
Chlorpyrifos (OP)	*Lu. longipalpis*	25.0 μg/ml (30 min)
		20.0 μg/ml (45 min)
	*P. papatasi*	30.0 μg/ml (60 min)
Fenitrothion (OP)	*Lu. longipalpis*	32.0 μg/ml (45 min)
		30.0 μg/ml (60 min)
	*P. papatasi*	30.0 μg/ml (60 min)
Malathion (OP)	*Lu. longipalpis*	40.0 μg/ml (60 min)
	*P. papatasi*	130.0 μg/ml (60 min)
Bendiocarb (CX)	*Lu. longipalpis*	6.0 μg/ml (40 min)
		5.0 μg/ml (60 min)
	*P. papatasi*	2.0 μg/ml (30 min)
		1.0 μg/ml (40 min)
Propoxur (CX)	*Lu. longipalpis*	3.0 μg/ml (30 min)
		2.0 μg/ml (40 min)
	*P. papatasi*	3.0 μg/ml (30 min)
		2.0 μg/ml (35 min)

^a^OP, organophosphate; CX, carbamate

**Table 3:** Diagnostic doses and diagnostic times for pyrethroid and DDT insecticides at time-to-knockdown and after 24-hours

Insecticide (Insecticide class^a^)	Species	Diagnostic dose and diagnostic time (for time-to-knockdown)	Diagnostic dose after 24 hours for mortality
Cypermethrin (PYR)	*Lu. longipalpis*	20.0 μg/ml (40 min)	20.0 μg/ml
		10.0 μg/ml (60 min)	
	*P. papatasi*	95.0 μg/ml (45 min)	60.0 μg/ml
		65.0 μg/ml (60 min)	
Deltamethrin (PYR)	*Lu. longipalpis*	45.0 μg/ml (35 min)	30.0 μg/ml
		15.0 μg/ml (40 min)	
		5.0 μg/ml (60 min)	
	*P. papatasi*	45.0 μg/ml (35 min)	25.0 μg/ml
		25.0 μg/ml (40 min)	
		15.0 μg/ml (45 min)	
		5.0 μg/ml (60 min)	
λ-Cyhalothrin (PYR)	*Lu. longipalpis*	4.0 μg/ml (40 min)	1.0 μg/ml
		3.0 μg/ml (45 mins.)	
		1.0 μg/ml (60 min)	
	*P. papatasi*	4.0 μg/ml (40 min)	6.0 μg/ml
		2.0 μg/ml (60 min)	
Permethrin (PYR)	*Lu. longipalpis*	15.0 μg/ml (30 min)	15.0 μg/ml
	*P. papatasi*	60.0 μg/ml (40 min)	55.0 μg/ml
		50.0 μg/ml (60 min)	
DDT (OC)	*Lu. longipalpis*	7.5 μg/ml (30 min)	650.0 μg/ml
	*P. papatasi*	7.5 μg/ml (30 min)	470.0 μg/ml

^a^PYR, pyrethroid; OC, organochlorine

### Organophosphates

Two diagnostic doses for *Lu. longipalpis* have been determined for chlorpyrifos: 20.0 μg/ml at 45 minutes and 25.0 μg/ml at 30 minutes. Only one diagnostic dose was determined for *P. papatasi* to chlorpyrifos: 30.0 μg/ml at 60 minutes. Both *Lu. longipalpis* and *P. papatasi* have identical diagnostic doses and diagnostic times for fenitrothion: 30.0 μg/ml at 60 minutes. *Lutzomyia longipalpis* has an additional diagnostic dose for fenitrothion of 32.0 μg/ml at 45 minutes. For malathion however, the diagnostic doses between species are markedly different. *Lutzomyia longipalpis*’ diagnostic dose is 40.0 μg/ml at 60 minutes, and *P. papatasi*’s diagnostic dose is 130.0 μg/ml at 60 minutes.

### Carbamates

Similar to the smaller lethal concentration (LC) values from Denlinger et al. [31], both *Lu. longipalpis* and *P. papatasi* have very small diagnostic doses. *Lutzomyia longipalpis* has a diagnostic dose and diagnostic time for bendiocarb of 6.0 μg/ml at 40 minutes or 5.0 μg/ml at 60 minutes. For propoxur, the diagnostic dose and diagnostic time is 3.0 μg/ml at 30 minutes or 2.0 μg/ml at 40 minutes. *Phlebotomus papatasi* has smaller diagnostic doses and diagnostic times for bendiocarb than *Lu. longipalpis*: 2.0 μg/ml at 30 minutes or 1.0 μg/ml at 40 minutes. For propoxur, the diagnostic dose is 3.0 μg/ml at 30 minutes or 2.0 μg/ml at 35 minutes, which is almost identical to the diagnostic dose and diagnostic time for *Lu. longipalpis*.

### Pyrethroids

*Phlebotomus papatasi* has a larger time-to-knockdown and 24-h mortality for cypermethrin than *Lu. longipalpis. Phlebotomus papatasi* has two time-to-knockdown diagnostic doses of 65.0 μg/ml at 60 minutes and 95 μg/ml at 45 minutes, and its 24-h mortality diagnostic dose is 60.0 μg/ml. Comparatively, *Lu. longipalpis*’ time-to-knockdown diagnostic doses are 10.0 μg/ml at 60 minutes and 20.0 μg/ml at 40 minutes, and its 24-h mortality diagnostic dose is 20.0 μg/ml. *Lutzomyia longipalpis* and *P. papatasi* have the same time-to-knockdown diagnostic doses of 5.0 μg/ml at 60 minutes and 45.0 μg/ml at 35 minutes. *Lutzomyia longipalpis* has an additional diagnostic dose of 15.0 μg/ml at 40 minutes, and *P. papatasi* has two additional diagnostic doses of 15.0 μg/ml at 45 minutes and 25.0 μg/ml at 40 minutes. Both species have almost equivalent 24-h mortality diagnostic doses to deltamethrin. *Lutzomyia longipalpis* requires 30.0 μg/ml and *P. papatasi* requires 25.0 μg/ml. Besides the carbamates, the time-to-knockdown diagnostic doses for lambda(λ)-cyhalothrin are the lowest for all ten insecticides. Both *Lu. longipalpis* and *P. papatasi* have a diagnostic dose of 4.0 μg/ml at 40 minutes. *Lutzomyia longipalpis* has two additional diagnostic doses of 1.0 μg/ml at 60 minutes and 3.0 μg/ml at 45 minutes. *Phlebotomus papatasi* has one additional diagnostic dose of 2.0 μg/ml at 60 minutes. Noticeably, *P. papatasi* has a lambda(λ)-cyhalothrin 24-h mortality diagnostic dose of 6.0 μg/ml, while it only required 1.0 μg/ml to cause 100 % mortality after 24 hours for *Lu. longipalpis*. For permethrin, *P. papatasi*’s time-to-knockdown diagnostic doses are 50.0 μg/ml at 60 minutes and 60.0 μg/ml at 40 minutes, and *Lu. longipalpis* has a diagnostic dose of 15.0 μg/ml in 30 minutes. There is a large difference between the two sand fly species permethrin 24-h mortality diagnostic doses: 55.0 μg/ml and 15.0 μg/ml for *P. papatasi* and *Lu. longipalpis*, respectively.

### Organochlorine

Both *Lu. longipalpis* and *P. papatasi* have small time-to-knockdown diagnostic doses of 7.5 μg/ml at 30 minutes when exposed to DDT. However, both species required very large 24-h mortality diagnostic doses: 650.0 μg/ml of DDT was needed for *Lu. longipalpis* and 470.0 μg/ml of DDT for *P. papatasi*.

## Discussion

The objective of this study was to demonstrate that the CDC bottle bioassay can be used to determine diagnostic doses and diagnostic times for phlebotomine sand flies to pyrethroid, organophosphate, carbamate and organochlorine insecticides. This work strengthens the collection of diagnostic doses and diagnostic times that are available for sand flies using the CDC bottle bioassay by presenting for the first time concentrations and times for *Phlebotomus* spp. [20, 32, 33]. The present study provides precise time-to-knockdown diagnostic doses for all ten insecticides for both sand fly species. In addition, for the first time, diagnostic doses for the 24-h recovery period are presented for sand flies to four pyrethroids and DDT.

There have been few studies that have determined diagnostic doses and diagnostic times for *Lu. longipalpis* using the CDC bottle bioassay. With the results presented in this study, comparisons can now be made for the insecticides malathion, deltamethrin and lambda(λ)-cyhalothrin. For our *Lu. longipalpis* colony, a dose of malathion of 40.0 μg/ml caused 100 % mortality in 60 minutes, while Marceló et al. [33] determined a concentration of 75.0 μg/ml caused 100 % mortality in 25 minutes. Against our colony of *Lu. longipalpis*, 45.0 μg/ml deltamethrin was needed to cause 100 % mortality in 35 minutes compared to 10.0 μg/ml in 35 minutes [33]. All currently published studies for lambda(λ)-cyhalothrin have found *Lu. longipalpis* to have low diagnostic doses. In the present study, a dose of 4.0 μg/ml was sufficient to cause 100 % mortality in 40 minutes. A dose of 15.0 μg/ml caused 100 % mortality in 30 minutes [33], and Santamaría et al. [32] found 10.0 μg/ml to cause 100 % mortality in approximately 60 minutes, although only three doses were tested and no precise diagnostic time was provided.

The only direct comparison that can be made for *Lu. longipalpis* is for deltamethrin as both colonies (present study and [33]) had equal diagnostic times of 35 minutes. Our colony needed 45.0 μg/ml to cause 100 % mortality, while *Lu. longipalpis* from [33] only needed 10.0 μg/ml. The CDC bottle bioassay protocol designates that a diagnostic dose needs to cause 100 % mortality in the 30 minute – 60 minute window of exposure (specifically at 30, 35, 40, 45 and 60 minutes) [8]. Some of the diagnostic times determined from Henriquez et al. [20] and Marceló et al. [33] for *Lu. evansi* and *Lu. longipalpis* do not fall into this window, and we are therefore not able to make direct comparisons. Future studies using the CDC bottle bioassay need to have comparable diagnostic times to be able to compare diagnostic doses between different populations of a sand fly species.

In accordance with Brogdon & Chan [8], as small as 5 μg/ml dose increments were used initially when determining diagnostic doses. It was necessary for lambda(λ)-cyhalothrin, fenitrothion, bendiocarb, propoxur and DDT, to work in increments as small as 1.0 μg/ml, 2.0 μg/ml, or 2.5 μg/ml because increments of 5.0 μg/ml were too large to effectively determine appropriate diagnostic doses. The small dose increments ensure that diagnostic doses are precise. An innacurate diagnostic dose that is too low in concentration has the potential of displaying false-positives of resistance because individuals will survive during the bioassay. An innacurate diagnostic dose that is too high will potentially display false-negatives of resistance because resistant individuals will be killed even if they are demonstrating a quantifiable level of resistance [8].

One potential limitation of this study was the use of 1,000-ml bottles and not the standard 250-ml bottles [7, 8]. There are though, published examples of non-standard volume bottles that have been used to assess insecticide susceptibility and determination of diagnostic doses and diagnostic times with the CDC bottle bioassay [12, 31]. The 1,000-ml bottles described in this study are the same bottles used in Denlinger et al. [34]. With this increase in volume though, we were unable to use a larger quantity of flies in each bottle (> 10–25 of the required number of flies per 250-ml bottle [8]) due to the substantial sand fly demand needed from the lab colonies throughout the entirety of the experiment. The use of the same number of required flies (10–25) in the larger sized bottles potentially may have influenced the diagnostic doses that we observed. Despite an equivalent concentration of insecticide, a smaller density of sand flies exposed per bottle volume (10–25 flies/1,000-ml bottle compared to 10–25 flies/ 250-ml bottle) and/or potential differences in air volume to bottle surface area may be a factor in the determination of the calculated diagnostic doses and diagnostic times. However, the ten insecticides used are contact insecticides, and the sand flies were regularly observed to be in contact with the interior surface of bottle due to them being poor fliers. The authors suggest that the diagnostic concentrations and times would be very similar for sand flies, regardless of these limited volume differences.

Diagnostic doses and diagnostic times of insecticides for susceptible populations of vector species are fundamentally required when assessing resistance in field populations [39–43]. Accordingly, the diagnostic doses and diagnostic times presented in this study should be used as an initial reference point for determining diagnostic doses and diagnostic times for other insecticide-susceptible populations. The criteria also differ between the WHO exposure kit bioassay and the CDC bottle bioassay. The most recent criterion for resistance for mosquito vectors by the WHO [11] states that resistance is present if there is less than 90 % mortality, while the criterion for resistance by the CDC states that resistance is present if there is less than 100 % mortality [8]. Using the CDC bottle bioassay to test mosquito populations for resistance, there are examples of employing both the WHO’s criterion for resistance [40, 44–47] and the CDC’s criterion for resistance [48, 49]. Recommendations from Saeidi et al. [24] suggest tailoring the WHO’s resistance criterion for sand flies because of the physiological, behavioral and size differences between mosquitoes and sand flies. We suggest that if the CDC bottle bioassay is used to assess sand fly insecticide susceptibility status, established diagnostic dose and times specific to sand flies and the CDC’s criterion for resistance should be used.

One important aspect of the CDC bottle bioassay is the 24-h holding period used for pyrethroids and DDT to allow insects to recover from “knockdown” [39, 41, 44, 50–52]. An imperative question with the CDC bottle bioassay is to determine which mortality endpoint to use when assessing resistance: at the time-to-knockdown or at the of the 24-h mortality [53, 54]. Both the knockdown endpoint and the 24-h mortality endpoint communicate different resistance mechanisms: knockdown resistance (*kdr*) via target-site insensitivity or metabolic detoxification. *Kdr* will cause knockdown to be lower than mortality, but metabolic detoxification resistance can cause mortality to be lower than knockdown [53]. Without the 24-h recovery period, the CDC bioassay could miss evidence of metabolic resistance because the lack of a 24-h recovery period does not allow resistant insects to recover; they may be scored as dead during the time-to-knockdown but would have recovered if allowed the 24-h recovery period [53]. In our experiments, the importance of the 24-hour recovery period as part of the CDC bottle bioassay protocol is evident for DDT. The time-to-mortality diagnostic doses were 63–87-fold greater than the time-to-knockdown diagnostic doses for *P. papatasi* and *Lu. longipalpis*, respectively (Table 3). This demonstrates that while sand flies may have small time-to-knockdown diagnostic doses, large concentrations are need to cause 100 % mortality after 24 hours.

The CDC bottle bioassay and WHO exposure kit bioassay are mutually used to detect insecticide resistance. However, a literature search of other studies conducted by [53] found differences in agreement between the two assays in detecting resistance in mosquitoes at both the time-to-knockdown and after 24 hours both at the 90 % and 98 % mortality cutoffs. Several studies have utilized the WHO exposure kit bioassay to assess insecticide resistance in sand flies [19, 21–27]. If future monitoring of insecticide resistance in sand fly populations is to utilize the CDC bottle bioassay, there will need to be a calibration of both the WHO exposure kit bioassay and CDC bottle bioassay. A synchronization of the diagnostic doses and diagnostic times for both assays will need to use the same population of sand flies, such that the same level of mortality can be derived from each assay [53].

The CDC bottle bioassay has been used for many years to track the spread of insecticide resistance in mosquitoes; however, this assay does not assess the intensity of insecticide resistance [54]. The CDC bottle bioassay intensity rapid diagnostic tests (I-RDT’s), developed by Bagi et al. [54], follows the CDC bottle bioassay protocol but measures insecticide concentrations 1×, 2×, 5× and 10× the known diagnostic doses. The intended goal is not so much with understanding the prevalence of insecticide resistance, but to quantify the intensity of resistance [54]. For sand flies, I-RDT’s are not yet necessary because the prevalence of resistance is low and baseline data from field collections are limited. Resistance prevalence for sand flies may be initially low because it has not been assessed very frequently or because it may not be very prevalent [13, 31, 55]. Regardless, knowing the speed with which resistance has developed and spread in mosquito populations demonstrates the need to continue to assess insecticide resistance prevalence in sand fly populations and to prepare I-RDT’s in areas where resistance is already present. The diagnostic doses and diagnostic times presented in this study provides necessary baseline data for developing CDC bottle bioassay I-RDT’s for sand flies.

## Conclusions

Evidence of insecticide resistance in worldwide populations of phlebotomine sand flies is a threat to the success of control programs that aim to mitigate the spread of leishmaniasis. It is crucial to have timely insecticide susceptibility data for different sand fly populations. The CDC bottle bioassay is one method to assess insecticide resistance, but it has been used infrequently with sand flies. With the diagnostic doses and diagnostic times presented here, the CDC bottle bioassay has great potential to be assimilated into sand fly control programs where other resistance-assessing methods are not feasible. The data presented in this study can serve as starting points for determining the susceptibility of field-collected and laboratory-reared *Lu. longipalpis* and *P. papatasi*, and for determining diagnostic doses and diagnostic times for other sand fly species of public health concern. Knowing if a population of sand flies is resistant to an insecticide or insecticide class is critical because it allows control strategies to be effectively implemented while not exacerbating the prevalence of insecticide resistance.

### Ethical considerations

The maintenance of SKH1 hairless mice (Charles River, Wilmington, MA, USA) and the experimental animal-use protocol was approved by Utah State University’s Institutional Animal-Care and Use Committee.
